# Bevacizumab for the Management of Pediatric Radiation Necrosis: a Narrative Review

**DOI:** 10.1007/s11912-026-01747-w

**Published:** 2026-02-05

**Authors:** Rimsha J. Afzal, Zackary Tareky, Raquel Rudy, Steven P. Howard, Nicholas Pytel, Brett Morris, Mustafa M. Basree

**Affiliations:** 1 Kentucky College of Osteopathic Medicine, Pikeville, KY USA; 2 Henry Ford Warren Hospital, Warren, MI USA; 3 Department of Human Oncology, University of Wisconsin School of Medicine and Public Health, Madison, WI USA; 4 Division of Pediatric Hematology/Oncology and Bone Marrow Transplant, School of Medicine and Public Health, Madison, WI USA

**Keywords:** Pediatric radiation necrosis, Radiation oncology, Bevacizumab, Vascular endothelial growth factor (VEGF), Central nervous system tumors

## Abstract

**Purpose of Review:**

Radiation necrosis (RN) presents a significant challenge in the treatment of pediatric central nervous system tumors. This narrative review synthesizes current evidence on the use of bevacizumab (BEV) for managing pediatric RN, drawing from case reports, retrospective reviews, and prospective clinical trials. While high-quality prospective data remain limited, emerging evidence suggests that BEV may be effective in alleviating both clinical symptoms and radiographic manifestations of RN in pediatric patients.

**Recent Findings:**

This review explores the pathophysiology of RN, BEV’s mechanism of action, and existing evidence on the use of BEV in pediatric RN. Emerging evidence suggests that bevacizumab may provide symptomatic relief and radiographic improvement in pediatric patients with RN.

**Summary:**

By addressing current knowledge gaps, the aim is to provide insight into the potential role of BEV in improving outcomes for pediatric patients with RN.

## Introduction

Brain and other central nervous system (CNS) tumors are the second leading cause of pediatric cancer, accounting for 17.3% of all new cases [1]. According to 2013–2017 data from the Surveillance, Epidemiology, and End Results (SEER) program, there are 3.2 new cases per 100,000 children per year, with median age of diagnosis at 8 years [1]. Pediatric cancer has a five-year relative survival rate of 74.9[1], with cancer being the second leading cause of death in children aged 1–14 years, after accidents [2]. While the cumulative therapeutic effect of radiation to tumors of the brain and CNS plays a crucial role in improving outcomes by controlling tumor growth, it carries the risk of delayed radiation necrosis (RN) affecting both irradiated and surrounding healthy tissues.

### Radiation Necrosis (RN)

Radiation therapy treats cancer by inducing DNA damage and triggering a local inflammatory response that resolves over time. However, in some cases, this inflammatory response, also referred to as RN in this context, persists and transforms into a chronic wound-healing-like process, leading to the recruitment of activated microglial cells that fail to repair the damage adequately [3]. The etiology of RN is multifactorial but is hypothesized to result from global disruption of the tissue matrix, vasculature, and endothelial structures, impairing the healing process [4, 5]. RN adversely affects the tissue microenvironment, influencing both molecular and histopathological processes [4–7].

The incidence of RN is primarily dose- and volume-dependent, with increased occurrence at extreme radiation doses and volumes [5–7]. A review of adult studies estimated an RN incidence rate of 5–10% at high radiation doses [8]. Risk of RN often limits therapeutic options in cases where re-irradiation is warranted to achieve disease control. RN is well-described and characterized in adults, but is less clearly defined in the pediatric population [9]. One reason for this is that pediatric tumor biology can differ compared to adults. For example, the WHO grading system, which is prognostically relevant in adults, has limited use in the pediatric population. Indeed, pediatric diagnoses were not formally included in WHO CNS classification until 2021[10, 11]. Nonetheless, pediatric radiation necrosis (PRN) is assumed to follow a similar biological and clinical course as adult RN.

Animal models suggest that pediatric patients may experience greater CNS radiation related changes compared to adults, as radiation therapy (RT) may disrupt brain and neuronal development [3, 12, 13]. Consequently, proton beam radiotherapy (PRT) has gained prominence in pediatric CNS management to limit normal brain tissue exposure. Retrospective [14] and prospective [15, 16] data demonstrate that PRT in pediatrics offers better target volume coverage, reduced organ-at-risk (OAR) exposure (e.g., minimize risk of symptomatic brain stem toxicity [17]), and a lower incidence of secondary in-field primary tumors compared with external beam radiation therapy (EBRT). However, despite its advantages, PRT remains associated with a considerable risk of RN.

Retrospective review of 60 pediatric patients treated with PRT for primary brain tumors reported an overall RN incidence of 31% (7.7% symptomatic), with a median onset of 5 months (range, 3–11 months) post-treatment [18]. Interestingly, specific radiation dose, tumor location, and surgical extent were not associated with RN risk, whereas concomitant chemotherapy (> 3 agents) and atypical teratoid rhabdoid tumor (ATRT) pathology were significant predictors. Moreover, RN was identified as a poor overall survival (OS) prognostic factor in 162 pediatric patients with diffuse intrinsic pontine glioma (DIPG), with an associated hazard ratio (HR) of 1.5 (95% confidence interval [CI], 1.0–2.2; *p* = 0.035)[19].

The gold standard for the diagnosis of RN is a biopsy to differentiate it from tumor progression [20, 21], however, clinical diagnosis is often relied upon in practice [22]. Advanced imaging techniques are emerging as potential non-invasive diagnostic tools. Hyperpolarized [13]C MR metabolic imaging of pyruvate has shown promise as a future standard due to its non-invasive nature [23]. MRI findings, such as T1 post-contrast “Swiss cheese” or “soap bubble” enhancement, are characteristic of RN[24]. Additionally, apparent diffusion coefficient (ADC) values from diffusion-weighted imaging (DWI) can help differentiate recurrence from RN, with high ADC values being predictive of necrosis [25]. MRI perfusion-weighted imaging (PWI) assessing cerebral blood volume (CBV) can further aid in distinguishing RN from tumor recurrence, as RN is often associated with hypoperfusion and low microvascular density [26]. The ratio of relative CBV in the area of concern to the contralateral normal tissue is a surrogate to estimate tissue microvascular density. Recurrent tumors typically have ratios of > 2.5, whereas in case of RN the ratio is < 0.6[26].

### Rationale and Mechanism of Action of Bevacizumab in Management of PRN

Bevacizumab (BEV) is a humanized monoclonal IgG1 antibody that inhibits vascular endothelial growth factor (VEGF) [27]. While initially developed for metastatic colorectal cancer, it has been utilized in managing patients with various solid tumors, as well as for RN in adults [27–30]. The pathogenesis of RN, and by extension PRN, is closely linked to vascular alterations. Radiation-induced vascular damage and resultant tissue hypoxia lead to increased hypoxia-inducible factor (HIF)−1α expression, which subsequently triggers VEGF release [31]. This process results in abnormal neovascularization, increased vascular permeability, and perilesional edema [31]. BEV counteracts these effects by inhibiting VEGF-mediated angiogenesis and vascular permeability, thereby reducing brain edema and limiting the progression of RN[31].

Preclinical data suggest that prophylactic BEV may ameliorate development of RN in animals [32, 33]. Aslan and colleagues examined 54 adult rats who underwent varying doses of radiation (Gamma Knife) and were treated with prophylactic BEV following completion of radiation [32]. Similar to human patients, increasing radiation dose led to higher incidence of RN. Receiving prophylactic BEV was associated with delayed onset RN irrespective of radiation dose. Interestingly, there was no protective role of BEV when initiated *after* rats developed symptoms. Anti-VEGF therapy may mediate its protective effects against RN by minimizing vascular malformation and protecting vessel density in irradiated tissues. The study also demonstrated that there were no MRI abnormalities noted in rat brains when BEV was administered without radiation exposure. Another preclinical study evaluated histological improvement in RN following a single dose of 50 Gy (Gamma Knife) [34]. They demonstrated that RN was not only improved with prophylactic BEV, but the degree of benefit was significantly higher when ramipril, an angiotensin-converting enzyme (ACE) inhibitor, was administered concurrently [34]. Finally, a brief report of two children with PRN who received 55 Gy in 5 Gy/week fractions following hemorrhage from arteriovenous malformations experienced both symptomatic improvements as well as radiographic improvement in vasogenic edema following administration of BEV [35]. Collectively, those studies provide an active area of research with the potential to improve clinical and quality of life outcomes in patients at high risk for development of RN.

### Evidence for using Bevacizumab in Management of PRN

Despite encouraging data, high-quality prospective studies evaluating BEV in PRN are lacking due to difficulties in patient accrual. The current knowledge is predominately derived from retrospective reviews and case studies (Table 1).

**Table 1:** List of studies describing pediatric patients with CNS disease treated with bevacizumab

**Author; year**	**Type of study**	**Disease**	**Location**	**Sample size**	**Age in years; (range)**
Castelli et al. 2023	Case report	LGG of pineal region	Frontal lobe	1	15
Kwong et al. 2021	Case report	Spetzler-Martin Grade 4 AVM	Right posterior medial frontal and anterior medial parietal lobes	1	6
Baroni et al. 2020	Retrospective	DIPG, LGG, HGG, PF-EPN-A, MB, CP, ATRT, ST-EPN-RELA, Chordoma	Brainstem, temporal lobe, frontal lobe, frontoparietal lobe	26	Average 10.7
Dahl et al. 2019	Pilot feasibility study	HGG, LGG	Brainstem, thalamus, pineal gland	7	Median 11; (4–15)
Dashti et al. 2015	Case report	Patient 1: Left posteriofrontal AVM. Patient 2: Spetzler-Martin Grade 2 AVM of corpus collusum	Posterior frontal lobe	2	11.5
Foster et al. 2014	Retrospective	LGG	Midbrain, thalamus, cervicomedullary junction	5	Median 15; (4–18)
Liu et al. 2009	Case series	Pontine Glioma	Pons	4	Unknown
Pillay Smiley et al. 2016	Case report	LGG	Brainstem	1	1.17
Plimpton et al. 2015	Retrospective	HGG, LGG	Thalamus, cerebral peduncle, suprasellar region	4	Unknown
Preuss et al. 2013	Case report	AVM	Choroid plexus	2	Median 9.4
Tan et al. 2017	Case report	sPNET	Frontoparietal lobe	1	15
Wang et al. 2012	Retrospective	AA	Thalamus	1	13

AA, anaplastic astrocytoma; ATRT, atypical teratoid rhabdoid tumor; AVM, arteriovenous malformation; CNS, central nervous system; CP, craniopharyngioma; DIPG, diffuse intrinsic pontine glioma; HGG, high-grade glioma; LGG, low-grade glioma; MB, medulloblastoma; PF-EPN-A, posterior fossa type A ependymoma; sPNET, supratentorial primitive neuroectodermal tumor; ST-EPN-RELA, RELA-fused ependymoma.

The most notable was a prospective single-arm trial (NCT01201850), designed to assess role of BEV in PRN, which was terminated in 2020 due to poor enrollment despite being open for 11 years. Patients between the ages of 1 and 25 years old with both neurologic deterioration and radiographic findings consistent with RN were to receive BEV (10 mg/kg IV) every 2 weeks for a total of 6 doses, with the primary outcome of completing at least 5 of the planned 6 doses. Out of planned accrual of 10 patients, 7 were enrolled and analyzed. Three out of the 7 patients completed BEV therapy, with 4 not completing due to disease progression [36]. BEV appears to have led to decreased gadolinium enhancement in all patients, decreased FLAIR signal in 5 patients, decreased tumor size in 5 patients, neurologic improvement in 4 out of the 7 patients, and numerically lower dexamethasone usage from mean of 6 mg (SD 4) pre-BEV to 2.3 mg (SD 5.2) post-BEV. There were no serious adverse events (AEs), with 4 patients experiencing other AEs including epistaxis (*n* = 1), mucositis (*n* = 1), hematologic (*n* = 5), and others. This study shows the challenge in accrual of a rare and unique patient population, though equally demonstrates a promising signal of efficacy. Moreover, a 2019 pilot feasibility study prospectively examined the use of BEV in seven patients aged 1–25 years with RN[37]. All patients exhibited radiographic improvement on T1-weighted imaging as well as improvement in neurologic exams; five of seven patients had experienced a decrease in the size of their tumor raising possibility of anti-tumor activity. Although patients in this study benefited following treatment, the study closed early due to poor accrual [37].

Liu et al. were the first to describe the use of BEV in four pediatric patients with diffuse pontine gliomas in 2009[38]. Those patients exhibited symptoms such as weakness, slurred speech, facial droop, ataxia, nausea, and vomiting. A decreased enhancement in the area of necrosis as well as symptomatic improvement following BEV was noted in three of the four patients, with one patient experiencing disease progression [38]. Since then, a growing number of retrospective studies and case reports highlight the effectiveness of BEV in reducing neurological symptoms and improving radiographic abnormalities (Table 2). A series of five patients with PRN (out of *n* = 101 pediatric patients; PRN rate of 5%) found that all exhibited clinical and radiographic improvements following administration of BEV [39]. In a cohort of 162 patients with DIPG, PRN was associated with worse survival and BEV (administered in 32 patients) was associated with numerically longer median OS compared to non-BEV patients, though statistical significance was not reached both in the overall cohort and those with PRN [19]. Among patients with PRN, median OS for BEV vs. without BEV was 13.3 vs. 11.4 months, respectively [19]. The authors concluded that there is possibly a role for BEV in patients with PRN.

**Table 2:** Characteristics of steroid and bevacizumab treatment

**Author; year; (n)**	**RT modality and dose** **Mean dose (range)**	**Post-RT time to RN development in months; (range)**	**Dose; range**	**# of doses**	**Median follow-up (months); range**	**Adverse effects from BEV**
Castelli et al. 2023	50.4 Gy/ 28F (n=1)	24	10 mg/kg every 4 weeks	24 cycles	12	Not reported
Kwong et al. 2021	SRS (16.9–18.5.9.5 Gy)	9	5 mg/kg	2 cycles	Not reported	Hypertonicity
Baroni et al. 2020; (n=26)	IMRT 54 Gy/30 (50.4–59.4.4.4/30-33F)	Median 3.8; (0.6–110.6)	10 mg/kg every 2 weeks (n=18); 5–10 mg/kg	Mean 4 doses: range (2–7)	Not reported	Grade 3 HTN (4%)
Dahl et al. 2019; (n=7)	IMRT 59.4 Gy/ 33F (n=5) 25 Gy/5F (n=2)	2.53; (0.7–9.7)	10 mg/kg every 2 weeks	6 doses (n=3); 3 doses (n=4)	4; (1.4–21.4)	Not reported
Foster et al. 2014; (n=5)	50.04 Gy/ 28 F (n=4) 55.20 Gy/ 28F (n=1)	Median 4.2; (1–11)	5–10 mg/kg, every 2 to 4 weeks	Median 6 (6–26)	Not reported	Avascular necrosis (40%)
Liu et al. 2009	54 Gy/ 30F (n=3)	Not reported	10 mg/kg every 2 weeks	5 n=2; 4 n=1; 3 n=1	10; 7; 5; 6	Not reported
Pillay Smiley et al. 2016	PBRT 46.8 Gy/ 26F	4	10 mg/kg every 2 weeks	Every 2 weeks for 12 doses, followed by 4 doses every 3 weeks, and a final 4 doses given every 4 weeks	12	Not reported
Dashti et al. 2015	SRS Mean 19.5 Gy	8	2.5 mg/kg	Not reported	Mean 8.5	Not reported
Plimpton et al. 2015	Mean 56.2 Gy (54–59.4.4 Gy)	Median 1.2; (0.5–8.5)	Not reported	Not reported	Median 13 (3–51)	Not reported
Preuss et al. 2013	55 Gy/ 5F weekly fractions	Not reported	5 mg/kg in 4 cycles every 2 weeks	4 cycles n=1; 11 cycles n=1	42 and 18	None
Tan et al. 2017	Not reported	Median 24 from RT; 3 from re-RT	7.5 mg/kg IV 3x weekly	5	11	Fatigue, TIA, paresthesia, loss of consciousness (6.67%)
Wang et al. 2012	56 Gy	Median 18; range not reported	7.5 mg/kg every 2 weeks	At least 2, total unknown	9	Not reported

BEV: Bevacizumab; Gy: Gray; HTN: Hypertension; IMRT: Intensity-Modulated Radiation Therapy; IV: Intravenous; PBRT: Proton Beam Radiation Therapy; RN: Radiation Necrosis; RT: Radiation Therapy; SRS: Stereotactic Radiosurgery; TIA: Transient Ischemic Attack.

Foster and colleagues reported on the use of BEV as either a primary therapy or as an adjunct to facilitate steroid tapering in five children with low grade gliomas (LGG) who developed symptoms and radiographic changes within 3 years of RT[40]. All five patients demonstrated both clinical and radiographic improvement following BEV initiation. However, two patients developed avascular necrosis, which was attributed to prolonged steroid use [40]. A separate case report described a 14-month-old infant with a brainstem LGG who developed RN with hydrocephalus four months after PRT to the cervical spine and brainstem [41]. The infant presented in critical condition with decreased consciousness and bradycardia but showed remarkable improvement within 48 h of BEV initiation. Additionally, tumor size decreased with complete resolution of RN, and steroids were successfully tapered off within one month. Given emerging evidence that BEV may have direct therapeutic effects on LGG, it is possible that the tumor size reduction observed in this case was partially due to an intrinsic antitumor effect [42, 43]. Complete steroid taper was also observed by Wang and colleagues in a thirteen-year-old with anaplastic astrocytoma, with improvement in performance status [44]. A separate case report described a 15-year-old male with a LGG of the pineal region who developed symptomatic PRN roughly 2 years after completing RT. The patient received 24 cycles of BEV and experienced sustained improvement in symptoms without recurrence at 1-year follow-up[45].

Furthermore, BEV has played a role in the management of pediatric cerebral arteriovenous malformations (AVM). In 2015, Dashti and collogues examined intraarterial anti-VEGF treatment for RN in two pediatric patients with cerebral AVM [45]. The first patient, a 12-year-old, presented with severe headaches and focal seizures affecting the right side of the body, while the second, an 11-year-old, exhibited weakness in the right upper and lower extremities. Both patients experienced symptom resolution following intraarterial BEV administration. MRI findings showed an 82% reduction in FLAIR signal in the first patient and a 27% reduction in the second within five months of treatment, further supporting the potential role of BEV in mitigating radiation-mediated vascular injury [45]. Another notable case is of a 6-year-old patient with Spetzler-Martin grade 4 AVM who had near-complete resolution of headaches and motor deficits within 2 months of BEV administration [46]. This patient had previously undergone stereotactic radiosurgery with an initial dose of 16.9 Gy, followed by reirradiation with 18.5 Gy.

The largest retrospective study on PRN, conducted by Baroni et al. in 2020, evaluated BEV use in 26 patients with various tumor histologies [47]. Half of the patients experienced neurological improvement after two cycles of BEV, while the remaining half demonstrated radiographic response. Interestingly, clinical and radiographic improvements did not always correlate. BEV was well tolerated, with only one patient experiencing grade 3 hypertension. The study found no significant correlation between BEV response and factors such as tumor histology, radiation dose, treatment location, drug dosage, or age at the time of treatment initiation. In 2016, Drezner and colleagues conducted a systematic review of 33 patients with PRN [48]. Among them, 11 received BEV in combination with steroids, with 10 showing both radiographic and symptomatic improvement.

The mechanism by which BEV alleviates PRN remains incompletely understood. While its anti-VEGF properties suggest that the primary benefit arises from reducing vascular permeability and associated edema, the literature also suggests a potential antitumor effect, making it difficult to distinguish between RN resolution and tumor regression in some cases. There are currently no high-quality, prospective studies specifically designed to elucidate the mechanistic role of BEV in PRN management. The HERBY trial (NCT01390948), conducted from 2011 to 2015, enrolled 121 pediatric patients (aged 3–18 years) with newly diagnosed high-grade gliomas (HGG) to evaluate the impact of BEV on event-free survival (EFS) [49]. Although not designed specifically to assess PRN, this randomized, open-label phase 2 trial compared standard-of-care postoperative RT with concomitant and adjuvant temozolomide (TMZ; *n* = 59) versus RT + TMZ plus BEV (*n* = 62). The trial found no improvement in EFS with the addition of BEV. There was no difference in the incidence of grade 3–5 adverse events (AEs) between treatment arms, though more patients in the BEV group discontinued therapy due to AEs [49]. A post-hoc analysis examined baseline radiographic characteristics in HERBY participants, identifying several predictors of clinical outcomes [50]. Specifically, tumors that were well-defined, unifocal, and centrally located with large necrotic and/or cystic volumes were associated with improved EFS and OS, *p* < 0.05. Pseudoprogression was observed in 7.2% of cases (8 out of 111 patients), with three cases occurring in the BEV group.

While the current literature suggests BEV may offer clinical benefit in PRN, several unique implementation challenges in this population warrant consideration. Intravenous access can be difficult, often requiring interventions and coordination with child life services. Additionally, BEV use is often an exclusionary criterion for certain clinical trials, prompting some families and providers to opt for corticosteroids instead to preserve trial eligibility. Lastly, given the rarity of pediatric CNS tumors, smaller centers may lack familiarity with BEV administration and follow-up, potentially limiting broader adoption outside of tertiary care institutions.

## Conclusion: should Bevacizumab be the Standard of Care for PRN Management?

Although high-quality prospective data are limited, a growing body of observational evidence suggests that BEV is likely safe and effective in managing PRN. Its use has been associated with symptomatic relief, radiographic response, and steroid-sparing effects, particularly in refractory or high-risk cases. However, practical barriers – including intravenous access challenges, exclusion from clinical trials, and limited experience at smaller centers – may limit its broader use. Prospective pediatric studies are needed to clarify its mechanism, define optimal indications, and guide standardized use in clinical practice.

## Key References

- Alrasheed AS, Aleid AM, Alharbi RA, et al. Brainstem Toxicity Following Proton Beam Radiation Therapy in Pediatric Brain Tumors: A Systematic Review and Meta-Analysis. Cancers (Basel). 2024;16(21):3655. Published 2024 Oct 30. doi:10.3390/cancers16213655.
  - This study demonstrates that the use of PRT in pediatrics offers better target volume coverage, reduced organ-at-risk (OAR) exposure, and minimizaes the risk of brainstem toxicity.
- Kim HJ, Lee JH, Kim Y, et al. Suggestions for Escaping the Dark Ages for Pediatric Diffuse Intrinsic Pontine Glioma Treated with Radiotherapy: Analysis of Prognostic Factors from the National Multicenter Study. *Cancer Res Treat*. 2023;55(1):41–49. doi:10.4143/crt.2021.1514.
  - This retrospective analysis in pediatric patients with DIPG portrayed radiation necrosis as an independent prognostic factor for overall survival. Notably, patients who received bevacizumab for managing RN had a slightly longer median overall survival.
- Shibahara D, Tanaka K, Togao O, et al. Bevacizumab for Brain Radiation Necrosis in Patients With Nonsquamous Nonsmall Cell Lung Cancer. *Clin Lung Cancer*. 2024;25(6):581–586.e3. doi:10.1016/j.cllc.2024.06.010.
  - This report details four clinical cases of bevacizumab to manage RN in patients with nonsquamous nonsmall cell lung cancer, demonstrating its potential as an effective interventation for symptomatic RN in non-primary brain tumors.
- Dashti SR, Kadner RJ, Folley BS, et al. Single low-dose targeted bevacizumab infusion in adult patients with steroid-refractory radiation necrosis of the brain: a phase II open-label prospective clinical trial. *J Neurosurg*. 2022;137(6):1676–1686. doi:10.3171/2022.2.JNS212006.
  - This study evaluates a single low-dose (2.5 mg/kg) targeted intra-arterial infusion of bevacizumab following steroid-refractory RN that reported durable clinical and radiographic imporvements in patients over a 12-month follow-up.
- Castelli B, Fonte C, Guidi M, et al. Corrigendum: Bevacizumab-Irinotecan combination therapy in recurrent low-grade glioma, previously treated with chemo-radiotherapy: a case report. Front Oncol. 2024;14:1372295. Published 2024 Feb 1. doi:10.3389/fonc.2024.1372295.
  - This case report in a pediatric patient with a recurrent, irradiated low-grade pilocytic astrocytoma achieved a durable one-year stable respons to combination therapy with bevacizuman and irinotecan.

## Data Availability

No datasets were generated or analysed during the current study.
